# Fracture Behavior of a Unidirectional Carbon Fiber-Reinforced Plastic under Biaxial Tensile Loads

**DOI:** 10.3390/ma17061387

**Published:** 2024-03-18

**Authors:** Kosuke Sanai, Sho Nakasaki, Mikiyasu Hashimoto, Arnaud Macadre, Koichi Goda

**Affiliations:** 1 Graduate School of Sciences and Technology for Innovation, Yamaguchi University, Ube 755-8611, Japan; sanaikousuke@gmail.com (K.S.); c005wcw@yamaguchi-u.ac.jp (M.H.); 2 Department of Mechanical Engineering, Yamaguchi University, Ube 755-8611, Japan; macadre@yamaguchi-u.ac.jp

**Keywords:** carbon fibers, epoxy resin, unidirectional laminate, biaxial test, fracture mode

## Abstract

In order to clarify the fracture behavior of a unidirectional CFRP under proportional loading along the fiber (0°) and fiber vertical (90°) directions, a biaxial tensile test was carried out using a cruciform specimen with two symmetric flat indentations in the thickness direction. Three fracture modes were observed in the specimens after the test. The first mode was a transverse crack (TC), and the second was fiber breakage (FB). The third mode was a mixture mode of TC and FB (TC&FB). According to the measured fracture strains, regardless of the magnitude of the normal strain in the 0° direction, TC and TC&FB modes occurred when the normal strain in the 90° direction, $\varepsilon_{y}$, ranged from 0.08% to 1.26% (positive values), and the FB mode occurred when $\varepsilon_{y}$ ranged from −0.19% to −0.79% (negative values). The TC&FB mode is a unique mode that does not appear as a failure mode under uniaxial tension; it only occurs under biaxial tensile loading. Biaxial tensile tests were also conducted under non-proportional loading. The result showed three fracture modes similarly to the proportional loading case, each of which was also determined by the positive or negative value of $\varepsilon_{y}$. Thus, this study reveals that the occurrence of each fracture mode in a unidirectional CFRP is characterized by only one parameter, namely $\varepsilon_{y}$.

## 1. Introduction

Carbon fiber-reinforced plastics (CFRPs) are a composite material with excellent specific strength and stiffness, superior to those of other synthetic fiber-reinforced plastics; they are used in various industrial fields, such as aerospace, automobiles, construction materials, and so on. Needless to say, materials in practical use as structural members are inevitably subjected to multi-axial loading, and CFRPs are no exception. The failure phenomena of CFRPs under multiaxial stresses are complicated because the mechanical properties vary greatly depending on the direction of loading due to its strong orthogonal anisotropy and include many interactive damage modes, such as fiber breaks, matrix fracture, interfacial debonding, delamination, and fiber kinking [1,2,3,4,5,6,7]. The above modes interact in a complex manner, eventually leading to fractures in the CFRPs.

**Figure 1:**
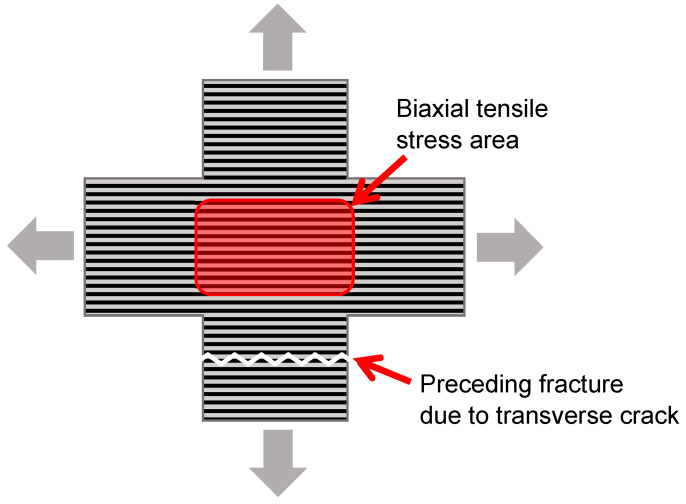
Schematic of fracture mode under T-T loading in a unidirectional CFRP (the fracture does not occur in the biaxial tensile stress area).

Although CFRPs are generally applied in a laminated form, representative data on their mechanical properties are published as results obtained from uniaxial tensile tests of a unidirectional form [8]. As is widely known, when unidirectional CFRPs are subjected to tensile loading in the direction of the fiber axis (0° direction), individual fiber breaks accumulate and eventually lead to material failure. In contrast, tensile loading in the fiber vertical axis (90° direction) results in transverse cracking, thereby bisecting the material. The question then arises: What kind of fracture behavior is observed when tensile loading occurs simultaneously in the 0° and 90° directions? Such fracture behavior should be of interest, but for some reason has not been discussed. The reason, the authors presume, is that although the biaxial tensile loading test has been successfully conducted on laminated CFRPs, it is difficult to perform for unidirectional CFRPs. According to the authors’ experience, the CFRP does not fracture in the region working on biaxial tensile stresses, but it does in the uniaxial loading portion along the 90° direction when the specimen shape is a cruciform, as shown schematically in Figure 1. In fact, there are very few papers that address biaxial tensile loading tests of unidirectional CFRPs in the 0° and 90° directions. On the other hand, various failure criteria [9,10,11,12,13] have been proposed for the fracture of unidirectional fiber composites, and many computational simulations [14,15] as well as continuum damage mechanics models [16,17,18,19] have been applied. Their validity has been confirmed by comparing experimental data, such as off-axial tests [9,10,12,13], axial tension/transverse compression biaxial loading [13], tension or compression/shear biaxial loading [13,19], transverse tension [20] or compression [21], axial tension and transverse compression [22], in-plane [23] or out-of-plane shear [24], and so on. However, there are only a few comparisons using biaxial tensile loading tests [13,19], and those are based on data from Soden et al. [25] for unidirectional E-glass/epoxy, with no comparison to CFRP. As with the aforementioned fracture behavior, there is currently insufficient information on stress and strain at fracture under biaxial tensile loading.

Cruciform specimens are often used to determine the mechanical properties of CFRPs under biaxial tensile loading. However, most of the papers have been conducted on CFRP laminates, such as cross-ply and angle-ply [26,27,28,29,30], and there are few papers that apply the cruciform specimens to unidirectional CFRPs. The only reported paper is the case where the specimen was tensile-loaded from two transverse directions perpendicular to the fiber axis [31]. Meanwhile, the shape of the biaxial test specimen has been studied according to the loading conditions as follows. Goto et al. [32] investigated the tensile strength of a unidirectional CFRP laminate in the 0° direction subjected to compressive loading in the 90° direction. The specimen used was dumbbell-shaped, but the two surfaces on both sides subjected to transverse compressive loading were simple planes. Rev et al. [33] proposed a thin-ply specimen composed of angle-ply and unidirectional CFRP, where an in-plane biaxial stress state of longitudinal tension and transverse compression is induced in the central 0° layers by the scissoring deformation of the angle-plies. The mechanism of transverse compressive loading differs from that of Goto et al., but the specimen also has a long strip shape in the 0° direction. Potter et al. [34] and Kang et al. [35] conducted a biaxial compression test on CFRP laminates. In this test, square-shaped specimens were used to apply compressive loads simultaneously from four directions. Thus, the specimen shape used for biaxial tests differs between tension–compressive loading (T-C loading) and compression–compressive loading (C-C loading). From such a background, the authors developed a unique unidirectional CFRP specimen for tension–tension loading (T-T loading) and succeeded in fracturing it in its biaxial tensile loading area [36].

The purpose of this study is to characterize the fracture modes of a unidirectional CFRP under proportional and non-proportional T-T loading, using the developed cruciform specimens. This study also aims to investigate the relationship between the fracture behavior and the strains in the 0° and 90° directions by measuring them at fracture. The results showed that three different fracture modes, namely transverse crack (TC), fiber breakage (FB), and TC including FB (TC&FB), were observed in both T-T loading tests. In addition, it was found that TC and TC&FB modes occurred when the strain in the 90° direction was positive, regardless of the difference in the two loading tests. However, the strains in the 90° direction (denoted as $\varepsilon_{y}$) causing the TC&FB mode, were clearly lower than those of the TC mode. This fact implies that the maximum strain criterion [37] is not applicable for the unidirectional CFRP under T-T loading. The strain range for the FB mode occurrence was also clarified, such that this mode occurred only when $\varepsilon_{y}$ was negative, despite the fact that the FB and TC&FB modes were comparable in strains in the 0° direction (denoted as $\varepsilon_{x}$). Thus, this study reports that the occurrence of each fracture mode is characterized by only one parameter $\varepsilon_{y}$.

## 2. Materials and Methods

### 2.1. Materials

The reinforcing material used was PAN-based carbon fiber tows (TR50S 12L and 15L supplied by Mitsubishi Chemical Corporation, Tokyo, Japan). The carbon fibers are hereafter abbreviated as CFs. For the matrix resin, epoxy EP-4901 (ADEKA Co., Ltd., Tokyo, Japan) was used as the resin base, and Jeffarmin T-403 (Huntsman International LLC, The Woodlands, TX, USA) was used as the hardener. The epoxy resin base and hardener were mixed at the manufacturer’s recommended mixing ratio of 100:46. The obtained mixture was degassed using a vacuum dryer and injected into the mold by hand, as described in Section 2.2. The properties of the CF tows and epoxy resin are shown in Table 1. The properties of the epoxy resin were obtained by tensile test in the authors’ laboratory.

### 2.2. Preparation Method of Cruciform Specimen

A cruciform specimen has been used for T-T loading tests on such laminates as cross-ply and angle-ply [26,27,28,29,30]. The present study applies this specimen to a unidirectional CFRP. According to these papers [26,27,28,29,30], conventional cruciform specimen shapes have a circular or square indentation referred to as ‘gauge area’ in the center. The authors also attempted to make this indentation and to fracture it at the center where the biaxial tensile load occurs, but it often broke prematurely at the arm. Through trial and error, we found that by embedding the CFRP laminae in the center of the resin outside the gage area and making it longer in the fiber direction, all fracture modes shown later occurred within the gauge area without arm fracture. Thus, the shape of the gauge area differs from that of the above papers.

**Figure 2:**
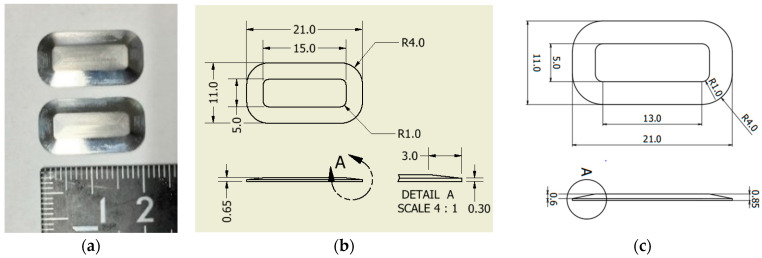
Convex parts to form gauge area. (**a**) Convex parts [36]; (**b**) shape and dimension of the convex part for 12L CF tow (unit: mm); (**c**) shape and dimension of the convex part for 15L CF tow (unit: mm). Note: The detail of A in (**c**) is the same as in (**b**).

In the design of the cruciform specimen, the thickness dimension of the gauge area was determined such that the specimen is fractured in the 0° direction within the load capacity of the biaxial testing machine used. In other words, since the number of CF tows is adjustable according to the capacity, the dimension can simply be calculated. The numbers of CF tows used were three for the 12L CF tow and two for the 15L CF tow, respectively. To achieve specimen preparation with such a gauge area, a pair of convex aluminum parts with flat top and bottom surfaces were used, as shown in Figure 2. The shape and aspect ratio of the convex parts are empirically optimal. In the gap between the two parts, resin pre-impregnated CF tows are aligned with physical restraints, as mentioned later. The gap distances between the two convex parts are designed to be 0.45 mm for the 12L CF tow and 0.3 mm for the 15L CF tow, respectively.

**Figure 3:**
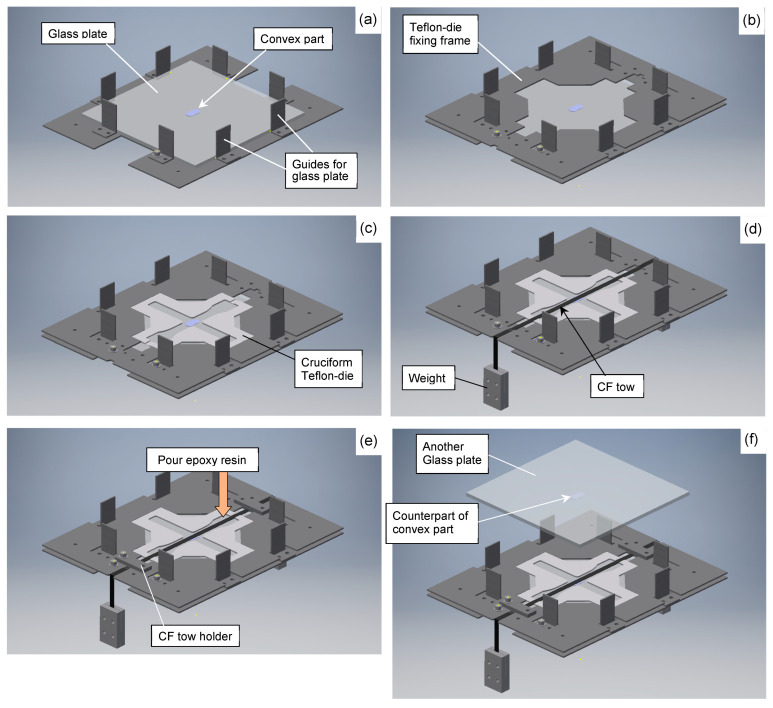
Schematic of cruciform specimen preparation procedure.(**a**) Set glass plate and position convex part in the center. (**b**) Set Teflon-die fixing frame. (**c**) Set cruciform Teflon-die. (**d**) Set pre-impregnated CF tows and fixation with weights. (**e**) Pour resin. (**f**) Set another glass plate. In (**f**), the counterpart convex part is attached to the back side of the glass plate.

**Figure 4:**
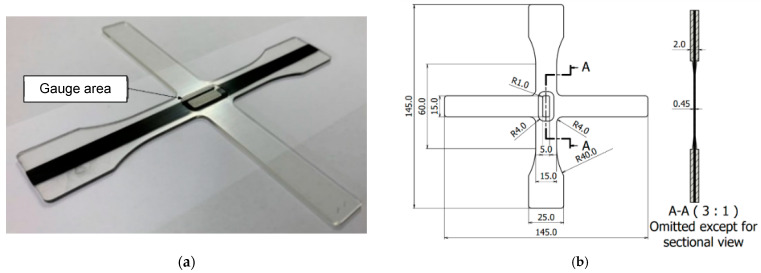
Cruciform specimen developed for the T-T loading test [36]. (**a**) Completed cruciform specimen; (**b**) shape and dimension used for the 12L CF tow (unit: mm). A-A bold line means cross section.

**Figure 5:**
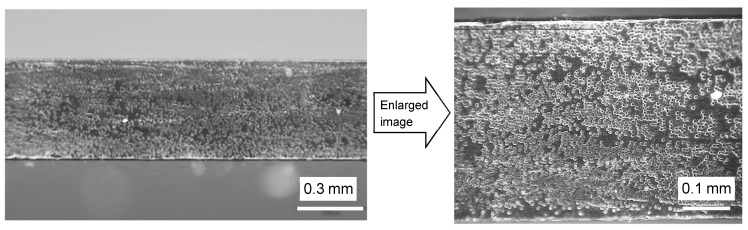
Transverse cross-section in gauge area of the cruciform specimen using the 12L CF tow.

The preparation method of the cruciform specimen is described in detail in the previous report [36], but this paper provides an outline. First, a convex part was glued to the center of the glass plate, which was placed into a stainless-steel frame with L-shaped metal fittings (Figure 3a). Then, a Teflon-die fixing frame was slid into them, and a Teflon-die cut out in a cruciform shape was placed into the frame, as shown in Figure 3b,c. Next, the prepared epoxy resin was impregnated to the CF tows. In order to place the CF tows in the center of the thickness direction, a 9.8 N weight was suspended at both ends to tension the CF tow, as shown in Figure 3d. This weight was adopted through several attempts in order to achieve a tension that did not loosen the CF tows and which was negligible in fracture load. During this process, air bubbles were generated, so the CF tows were degassed with a vacuum pump in the state shown in Figure 3d. After that, the epoxy resin was again poured into the Teflon-die (Figure 3e), and another glass plate, with the convex part of the counterpart shown in Figure 3f, was placed in the stainless-steel frame to avoid air bubbles. The entire apparatus, including the material before curing, was placed in a thermostatic dryer and cured for 24 h at 25 °C and post-cured for 6.5 h at 60 °C. The appearance of the resultant cruciform specimen is shown in Figure 4a, and its dimensions and shape are shown in Figure 4b. Figure 5 shows a typical fiber vertical cross-section of CFRP filled in the ‘gap’ described above. Although there is some variation in the fiber dispersion, it can be confirmed that large-scale resin-rich areas occurring between laminae are not found on the specimen surface due to the constraint by the convex parts.

Since the thickness of the CFRP cross-section is considered to be approximately equal to the gap distance described above, and the widths are both 7 mm, the fiber volume fractions are, respectively, calculated to be 44.0% and 55.0% for the 12L and 15L CF tows.

### 2.3. Stress Distribution

**Figure 6:**
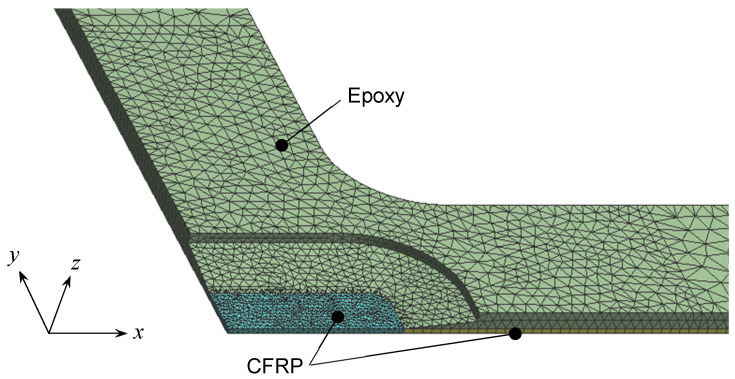
Finite element mesh applied for the cruciform specimen (CFRP: peacock blue area and bottom area under the epoxy. Epoxy: lime green area).

It was also demonstrated in reference [36] that the stress distributions in the fiber and fiber vertical directions at the gauge area are nearly uniform when using general-purpose finite element analysis software (ANSYS, ver. 18.0). The simulation results are summarized below. Figure 6 shows the element mesh of the gauge area and its vicinity used in the analysis, where the model shape is a 1/8 model in consideration of the symmetry of the specimen. The element type used is a linear tetrahedral element. The model consists of two areas, namely CFRP and epoxy areas, which constitute the cruciform specimen. The numbers of nodes and elements are 55,740 and 24,780, respectively. The material properties of CFRP were set as an orthotropic elastic material, and those of epoxy were chosen as an isotropic elastoplastic material. The material properties of the epoxy were based on the stress–strain diagram of the epoxy resin used for the specimens, and the multilinear approximate isotropic hardening law was applied for the plastic region. The interfacial contact between the CFRP and epoxy was assumed to be fully bonded, and the deformation perpendicular to the symmetry plane was constrained. The load boundary condition was applied at the ends of the specimen along *x*- and *y*-axes. The material constants used in the simulation are shown in Table 2.

**Figure 7:**
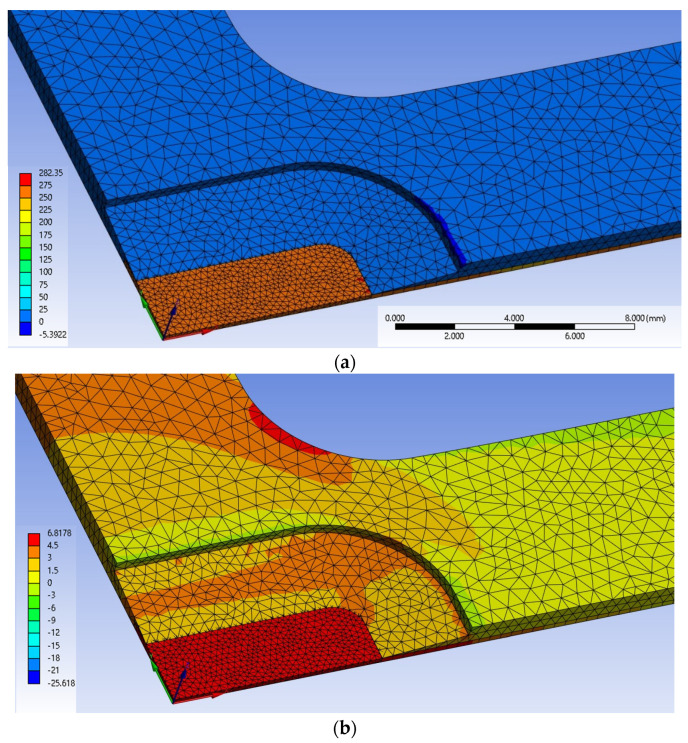
Simulation results of stress distributions, $\sigma_{x}$ and $\sigma_{y}$ (the unit of the numbers next to the color display is MPa, and the top and bottom are the maximum and minimum values, respectively). (**a**) Stress distribution of $\sigma_{x}$; (**b**) stress distribution of $\sigma_{y}\;$.

Figure 7a,b show the stress distributions, $\sigma_{x}$ and $\sigma_{x}$, along the *x*- and *y*-axes, respectively. In this simulation, the loads, 1000 N and 100 N, were, respectively, applied at the ends of the *x*- and *y*-axes as boundary conditions. Although some stress concentration occurred at the border between the flat CFRP and the inclined epoxy areas, the stress distributions in the gauge area were stable without extreme variation, as shown in both figures. Table 3 shows the ranges in the gauge area of the simulated maximum and minimum stresses under various boundary conditions. It is found from Table 3 that the ranges do not have a large variation. Thus, the cruciform specimen shown in Figure 4 was used.

### 2.4. Biaxial Tensile Test

**Figure 8:**
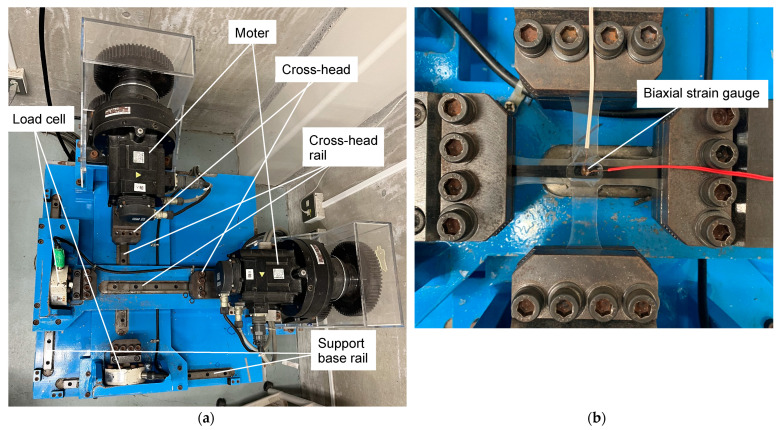
Biaxial tensile and compressive testing machine. (**a**) Top view of the biaxial tensile and compressive testing machine; (**b**) Top view after mounting the cruciform specimen attached with the biaxial strain gauge. This product consists of two uniaxial strain gages, which are placed perpendicularly to each other.

For the biaxial tensile test, a biaxial tension and compression testing machine (manufactured by Kisan Chemical Engineering & Works Co., Ltd., Yamaguchi Shunan, Japan, with a capacity of 10 kN in each axis) shown in Figure 8a was used. The machine has independent structures on the *x*-axis and *y*-axis, and is equipped with a motor and a load cell at the bases of both the *x*-axis and *y*-axis. The power of each motor is transmitted to each shaft through gears, and the *y*-axis base moves along the rail when the tensile or compressive load is applied to the *x*-axis, and vice versa. The load conditions were set by changing the displacement speed ratios of the *x*- and *y*-axes cross-heads. Table 4 shows the loading conditions applied by changing the cross-head speed. The U10 condition is a uniaxial tensile test using a cruciform specimen with the *y*-axis arm of the 90° direction cut off. On the other hand, it is known that the fiber end-face effect often appears in uniaxial tests in the fiber vertical direction. Hence, the loading condition on the *y*-axis that results in zero stress in the 90° direction was estimated in advance by the finite element analysis software, and the cruciform specimen was used as it is. B114 corresponds to this condition.

Non-proportional tests were also conducted in this study, in which several arbitrary loads were applied in the *x*-axis first; the loads were then fixed, and the *y*-axis loading started and continued up to the specimen’s fracture. This condition is denoted as X1Y2. Another condition, denoted as X2Y1, is the case when the order of loading is the reverse of X1Y2. Both of the cross-head speeds were set as 0.01mm/s.

To measure strains along the 0° and 90° directions, a biaxial superimposed strain gauge (KFGS-1-120- D16-11, Kyowa Electronic Instruments Co., Ltd., Tokyo, Japan) was attached to the center of the gauge area, as shown in Figure 8b. The measurement was carried out until the specimen was fractured.

## 3. Results and Discussion

### 3.1. Fracture Modes

**Figure 9:**
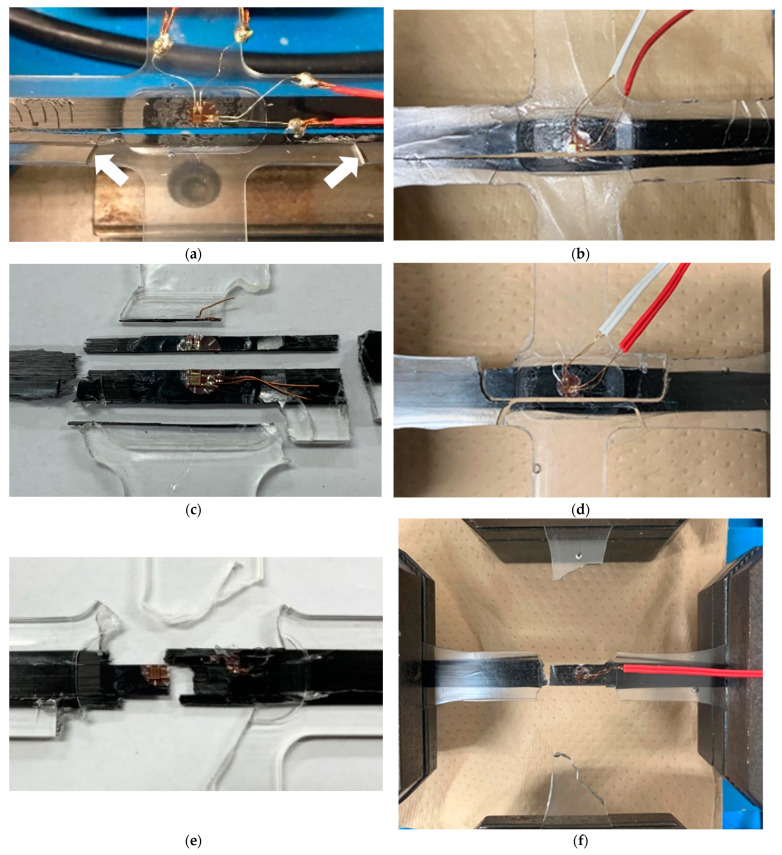
Various fracture modes under biaxial tensile loads of the cruciform specimens. (**a**) TC mode (B11) [36]; (**b**) TC mode (X1Y2); (**c**) TC&FB mode (B31) [36]; (**d**) TC&FB mode (X1Y2); (**e**) FB mode (B51) [36]; (**f**) FB mode (X2Y1). The arrows in (**a**) indicate resin cracks.

After the proportional and non-proportional T-T loading tests, the gauge areas of the fractured specimens were observed visually. It was found that there were three types of fracture modes. The first is the transverse crack (referred to as TC), in which a single crack runs along the fiber direction, as shown in Figure 9a,b. This fracture mode was observed in all specimens under B11, B12, B114 conditions, and in several X1Y1 conditions. In B11, there were several specimens in which the main crack branched and secondary cracks occurred in the resin, as shown by the arrows in Figure 9a. The second mode appeared such that the TC occurred within the gauge area but did not extend so much to the outside. In addition, cracks perpendicular to the fibers are observed at two locations just outside the gauge area, as shown in Figure 9c,d. This is undoubtedly a fracture mode due to fiber breakage (referred to as FB). This mode is referred to as TC&FB, which was observed in all specimens under B21 and B31 conditions, and under several X1Y2 conditions. The TC&FB mode is a unique mode that does not appear under uniaxial tension; instead, it only appears under biaxial tensile loading. The third one is only the FB mode, as shown in Figure 9e,f, which was observed in most of the specimens under B41 and B51 conditions and in all the specimens under U10 and X2Y1 conditions.

Table 5 shows the average fracture loads *F_x_* in the 0° direction and *F_y_* in the 90° direction for proportional loading conditions along with fracture modes. *F_y_* values of B41 and B51 showing the FB mode were lower than those in the other loading conditions, which suggests that the FB mode was mainly caused by *F_x_*. On the other hand, the *F_x_* of B31 was close to the *F_x_* of B41, and the *F_y_* of B31 was also close to the *F_y_* of B11 and B12. Such results imply that it is not clear which mode (FB or TC) occurs first in the FB&TC mode. Due to the capacity of the instrument used in this study, the strain measurement interval was set to 5.0 μs. Unexpectedly, at the time of fracture of the specimens, both strains on the *x*- and *y*-axes changed at the same time within the above interval. This fact means that it is difficult to distinguish which mode occurred first. Nevertheless, we believe that in the TC&FB mode, TC occurs first. As mentioned above, the FB in the TC&FB mode occurred at two outside locations where the thickness was larger than the gauge area, but there was no case where two FB modes appeared individually without the TC mode. Also, there was no FB mode at only one location outside the gauge area. If FB mode(s) occur first on the outside, such specimens without the TC mode should exist. However, the TC was always present in the gauge area. To rephrase the above, it is reasonable to conclude that in TC&FB mode, TC occurs prior to FB and the occurrence of TC triggers FB a moment later.

**Figure 10:**
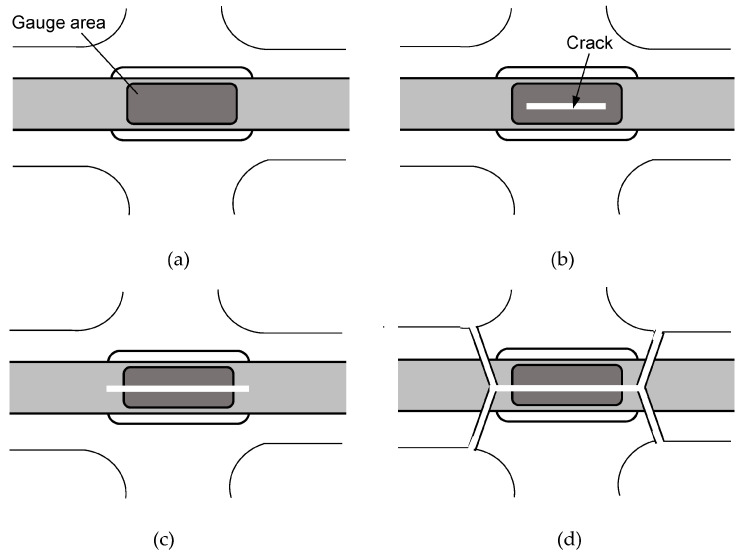
Schematic of the TC&FB formation process. (**a**) Initial state; (**b**) TC occurrence in the gauge area; (**c**) TC propagation outside the gauge area; (**d**) TC branching followed by two FB modes.

Thus, we suggest the formation process of the TC&FB mode, as shown in Figure 10. Since B21 and B31 conditions provide large loads in the 0° direction, local single fiber-breaks are able to occur, and subsequent fiber–matrix interfacial debonding easily occurs due to another loading in the 90° direction. As a result, although the fracture approaches the FB mode, such multiple localized debonding can also cause the TC mode even at smaller strain levels than B12 or B11. Once TC occurs, it propagates instantaneously, as shown in Figure 10a,b. After that, the crack propagates outside the gauge area at high speed, as shown in Figure 10c. In general, cracks of rigid polymers are known to branch not only under uniaxial tensile loading but also under biaxial tensile loading [39,40]. In this case, the crack also branches at the epoxy area. Finally, the tensile stress along the 0° direction greatly increases at the two branching locations, as shown in Figure 10d, where two FB modes occur instantaneously.

Table 6 shows the fixed loads and the average fracture loads for non-proportional loading conditions along with fracture modes. The fracture load *F_x_* of X2Y1* showing the FB mode is almost at the same level as those of the proportional condition, but the *F_x_* values of X2Y1 are lower than those in the proportional loading conditions, irrespective of the fixed load *F_y_*. This is because the number of tows is reduced from three to two in the specimen using the 15L CF tow. Since the ratio of the total numbers of monofilaments is 6:5 for the 12L CF tow to 15L, 6605 N and 6535 N of $F_{x}$ in the X2Y1 conditions are, respectively, estimated as 7926 N and 7842 N at the same number of monofilaments. Thus, we can say that these values correspond to fracture loads of the FB modes measured at the proportional loading.

TC&FB modes were obtained under several X1Y2 conditions, at which the fracture loads of $F_{y}$ are close to the fracture loads obtained under B21 condition. TC modes were also obtained under several X1Y2 conditions, at which the fracture loads of $F_{y}$ are also similar to the fracture loads under the B11 and B12 conditions. According to Reuss’ model and other models, Young’ modulus along the fiber vertical direction of a unidirectional lamina is insensitive to changes in the fiber volume fraction up to about 60% [41,42]. Therefore, the fracture loads showing TC and TC&FB modes may be at a similar level to those of proportional loading despite the different volume fraction, if the fracture mechanism of the two CFRP specimens is the same. In any case, it is concluded from the above results that the same fracture modes appear at similar fracture loads, regardless of the difference in the loading history.

### 3.2. Strain Histories and Fracture Strains

**Figure 11:**
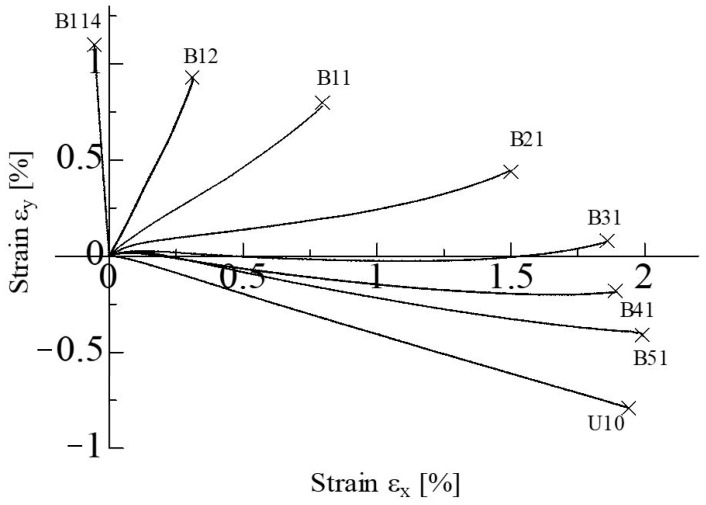
Typical $\varepsilon_{x}-\varepsilon_{y}$ diagrams under proportional biaxial loading. (The “×” in the figure indicates a fracture).

The results of the strain measurements of the unidirectional CFRP cruciform specimens under proportional T-T loading are shown in Figure 11. The horizontal and vertical axes, $\varepsilon_{x}$ and $\varepsilon_{y}$, in the figure are, respectively, strains along the 0° and 90° directions measured up to fracture. As can be seen from the $\varepsilon_{x}-\varepsilon_{y}$ curves of the B21, B31, B41, and B51 conditions, the specimens initially behave with a linear deformation and then change slightly to the positive side of $\varepsilon_{y}$ as they approach the final fracture. On the other hand, B141, B12, and U10 behave almost linearly up to fracture. The fracture modes were the TC mode for the B141, B12, and B11 conditions, and the TC&FB mode for the B21 and B31 conditions. The FB mode appeared with the B41, B51, and U10 conditions. Despite these differences in fracture modes, the slight changes in $\varepsilon_{y}$ in the B21, B31, B41, and B51 conditions are considered to be attributed to the nonlinearity of the matrix resin caused by tensile loading on the *y*-axis. This means that these are the conditions under which the matrix is greatly deformed plastically. In fact, when the neat epoxy resin used for the matrix was tensile-tested, plastic deformation was observed. Since the plastic work is stored in the specimen during deformation, it is inferred that a greater release energy is required to fracture the specimen, resulting in a complex fracture mode, such as TC&FB. It should be noted here that a positive $\varepsilon_{y}$ at the final fracture always shows the TC or TC&FB modes. That is satisfied even if the specimen exhibits a partially negative strain during the deformation process, as can be seen in B31. On the other hand, if the change in $\varepsilon_{y}$ remains negative, the fracture modes appeared as FB.

**Figure 12:**
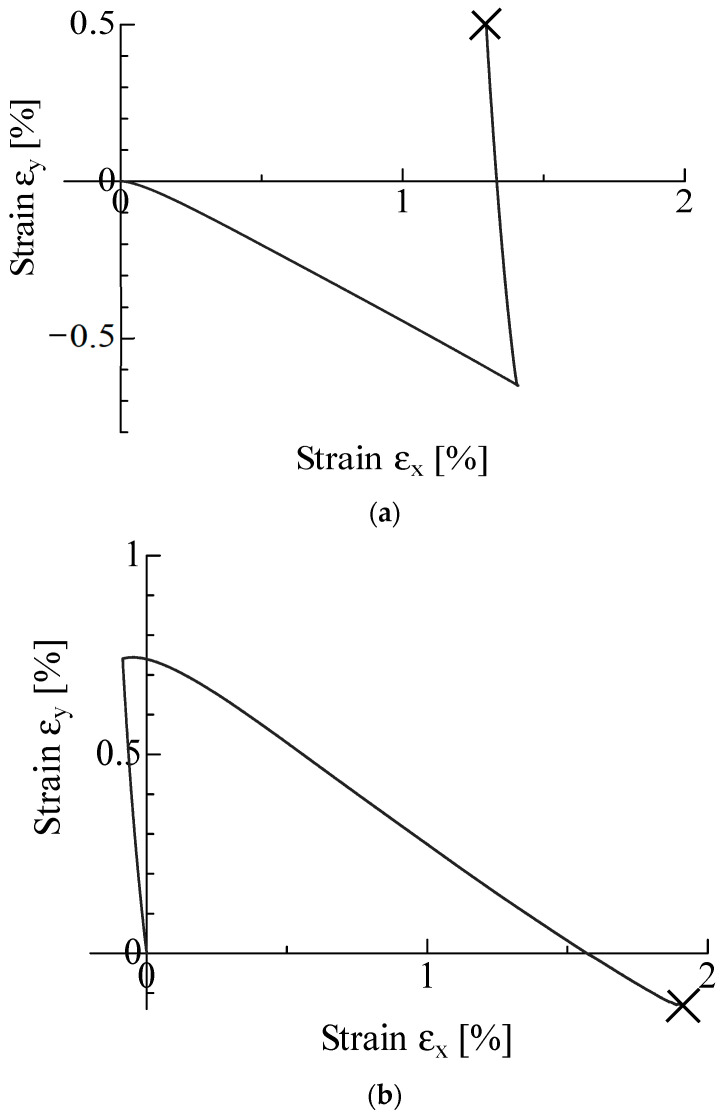
Typical $\varepsilon_{x}-\varepsilon_{y}$ diagrams under non-proportional biaxial loading. (**a**) X1Y2 condition; (**b**) X2Y1 condition. (The “×” in the figure indicates a fracture).

Typical $\varepsilon_{x}-\varepsilon_{y}$ diagrams under non-proportional T-T loading are shown in Figure 12a,b for the X1Y2 and X2Y1 conditions, respectively. The former condition caused the TC mode, while the latter resulted in the FB mode. In each figure, the initial deformation occurs in two directions due to the Poisson effect despite the fact that the load is first applied along a single axis. It is found from these figures and the observation results that the fracture mode is determined by the positive or negative value of $\varepsilon_{y}$, as in the proportional loading test.

**Figure 13:**
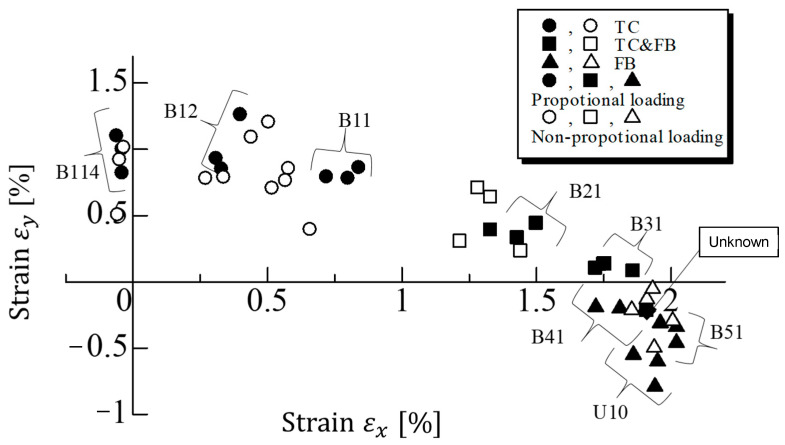
Fracture strains of cruciform specimens under proportional and non-proportional loading. Note: The number overlapping an open circle near the vertical axis is 0.5.

We next discuss the transition point from the TC&FB to FB modes through the fracture strain data. Figure 13 shows the fracture strains of the cruciform specimens under proportional T-T loading with filled symbols. It is confirmed that when the fracture strain $\varepsilon_{y}$ is in the range 0.60 to 1.26%, the fracture mode is the TC mode. The strains $\varepsilon_{y}$ causing the TC&FB mode are in the range of 0.08 to 0.50%. Although these strains are all positive, those of the TC&FB mode are given with smaller $\varepsilon_{y}$. This difference signifies that the maximum strain criterion [37] is not satisfied. On the other hand, when the strain $\varepsilon_{y}$ is in the range of –0.19 to −0.79%, the fracture mode is FB. All specimens of U10 showed the strain $\varepsilon_{y}$ as being less than −0.50%. The results of fracture strains derived from the non-proportional loading test are added to Figure 13 with open symbols. It can be seen that the strains are distributed in a similar range to the proportional loading case. It is also found that FB modes appear when $\varepsilon_{y}$ is negative, and TC and TC&FB modes occur when $\varepsilon_{y}$ is positive.

In general, the fiber breakage condition is significantly important, because it determines the load-bearing capacity of structural members. Although Hashin’s or the maximum stress failure criterion has been used as the condition in many theoretical models and numerical simulations [7,11,12,13,14,15,16], the above results newly show that $\varepsilon_{y}$ is the key point when the members are under biaxial tensile loading. In other words, even if the simulated stresses reach the fiber breakage condition, attention should be paid to the phenomenon that a positive $\varepsilon_{y}$ does not generate isolated FB modes, but rather TC&FB first.

Thus, the fracture modes depend on the positive or negative value of $\varepsilon_{y}$, irrespective of the loading conditions. This leads to the conclusion that the transition of the fracture modes occurs when $\varepsilon_{y}\;is\;0\%$. This is achieved without any difference in $\varepsilon_{x}$, as seen in the comparison of B31 and B41. Unlike C-T and T-C loading, T-T loading is a combined loading condition that mutually constrains deformation. Therefore, when high tensile loads are applied in the *x*-axis as in the B41 and B51 conditions, compressive deformation due to the Poisson effect is dominant in the 90° direction rather than tension. The $\varepsilon_{y}$ increases further in the negative direction as the fiber–axis tension continues; hence, the TC mode no longer occurs. The tensile load then continues to be applied, and eventually the FB mode occurs.

The evaluation of fracture stress in the gauge area was not carried out in this study, but this is an issue to be covered in the near future, along with the proposal of the fracture criterion of unidirectional CFRPs under T-T loading.

## 4. Conclusions

The failure behavior of unidirectional CFRPs subjected to biaxial tensile loading in the 0° and 90° directions has not been experimentally clarified prior to this study. Therefore, the fracture behavior of the specimens was investigated under various proportional and non-proportional T-T loading conditions using a new cruciform specimen for tension–tension loading (T-T loading) developed for a unidirectional CFRP. The results obtained are summarized as follows:

(1) Biaxial tensile fracture of the unidirectional CFRP is enabled by a cruciform specimen with a gauge area longer in the fiber direction than in the fiber vertical direction at the center. This is applicable regardless of proportional or non-proportional loading.
(2) There were three fracture modes in the specimens: a transverse crack (TC), fiber breakage (FB), and both modes (TC&FB) occurring simultaneously. The TC&FB mode is a unique mode that does not appear in the fracture modes under uniaxial tension (FB and TC), but only under biaxial tensile loading.
(3) Under several conditions of proportional loading, the failure loads in the 0° direction, indicating the FB and TC&FB modes, were almost identical. In another condition, the fracture loads in the 90° direction, indicating the TC and TC&FB modes, were close to each other. From the aspect of fractured specimens, it was finally inferred that the TC occurred before FB, and the occurrence of the TC triggered FB instantaneously.
(4) The TC and TC&FB modes occurred when the strain in the 90° direction, $\varepsilon_{y}$, was positive. On the other hand, the FB mode occurred when $\varepsilon_{y}$ was negative despite the fact that FB and TC&FB modes showed almost the same strains in the 0° direction. It was concluded that the occurrence of each fracture mode is characterized by only one parameter, namely $\varepsilon_{y}$.

## Figures and Tables

**Table 1:** CF tow [38] and matrix resin properties.

Constituent Material	Density [Mg/m^3^]	Filament Diameter [mm]	Tensile Strength [MPa]	Young’s Modulus [GPa]	Fracture Strain [%]	$\sigma_{0.2}$ [MPa]	$H^{\prime }$ [MPa]
CF tow	1.82	7	4900	235	-	-	-
Epoxy resin	1.16	-	65.2	3.2	5.0	48.0	447

$\sigma_{0.2}$: 0.2% proof stress, $H^{\prime }$: Linear plastic work hardening coefficient.

**Table 2:** Material properties of CFRP used for the 12L CF tow.

*E*_1_ = 112.8 [GPa]	*ν*_12_ = 0.38	*G*_12_ = 6.0 [GPa]
*E*_2_ = 6.6 [GPa]	*ν*_13_ = 0.38	*G*_13_ = 6.0 [GPa]
*E*_3_ = 6.6 [GPa]	*ν*_23_ = 0.49	*G*_23_ = 2.2 [GPa]

*E*: Young’s modulus, *ν*: Poisson’s ratio, *G*: shear elastic modulus. Subscripts 1, 2, and 3 correspond to the *x*-, *y*-, and *z*-axes, respectively.

**Table 3:** Ranges in the gauge area of the computed maximum and minimum stresses.

Applied Loads		Simulated Stresses	
$F_{x}$ [N]	$F_{y}$ [N]	$\sigma_{x}$ [MPa]	$\sigma_{y}$ [MPa]
50	100	3.7–6.8	6.6–10.4
100	100	17.7–20.5	6.6–10.1
200	100	45.5–48.1	6.6–9.6
400	100	101–103	6.4–8.5
800	100	210–212	6.1–6.6
1000	100	267–279	5.2–6.2
1400	100	377–380	3.5–5.8
1000	-	285–286	0.06–0.52

**Table 4:** Proportional biaxial tensile loading conditions.

Loading Condition	Cross-Head Speed		Cross-Head Speed Ratio
	*x*-Axis [mm/s]	*y*-Axis [mm/s]	
B11	0.01	0.01	1:1
B21	0.02	0.01	2:1
B31	0.03	0.01	3:1
B41	0.04	0.01	4:1
B51	0.05	0.01	5:1
B12	0.01	0.02	1:2
B114	0.01	0.14	1:14
U10	0.01	-	-

*x*-axis: 0° direction, *y*-axis: 90° direction.

**Table 5:** Average fracture loads along *x*- and *y*-axes under proportional loading.

Loading Condition	Number of Specimens	Fracture Loads [N]		Fracture Mode
		$F_{x}$	$F_{y}$	
B11	3	3292	835	TC
B21	3	5728	818	TC&FB
B31	3	7457	773	TC&FB
B41	3	7415	595	FB *
B51	3	8058	567	FB
B12	3	1608	825	TC
B114	3	235	685	TC
U10	3	7318	0	FB

CF tows used are all 12L; * one of the specimens is unknown.

**Table 6:** Average fracture loads along the *x*- and *y*-axes under non-proportional loading.

Loading Condition	Number of Specimens	Fixed Load [N]		Fracture Load [N]		Fracture Mode
		$F_{x}$	$F_{y}$	$F_{x}$	$F_{y}$	
X2Y1 *	3	-	460	7203	-	FB
X2Y1	1	-	610	6605		FB
X2Y1	1	-	420	6535		FB
X1Y2	1	5515	-		890	TC&FB
X1Y2	3	4500	-		807	TC&FB
X1Y2	2	2265	-		813	TC
X1Y2	7	2200	-	-	853	TC
X1Y2	2	500			790	TC

CF tow used is 12L for X2Y1 * only; it was15 L for all others.

## Data Availability

Data are contained within the article.
